# Evaluation of AAPM Reports 204 and 220: Estimation of effective diameter, water‐equivalent diameter, and ellipticity ratios for chest, abdomen, pelvis, and head CT scans

**DOI:** 10.1002/acm2.12223

**Published:** 2017-11-27

**Authors:** Christiane S. Burton, Timothy P. Szczykutowicz

**Affiliations:** ^1^ Department of Radiology University of Wisconsin‐Madison Madison WI USA; ^2^ Departments of Radiology, Medical Physics, and Biomedical Engineering University of Wisconsin‐Madison Madison WI USA

**Keywords:** AAPM Report 204, AAPM Report 220, effective diameter, ellipticity ratio, water‐equivalent diameter

## Abstract

**Purpose:**

To confirm AAPM Reports 204/220 and provide data for the future expansion of these reports by: (a) presenting the first large‐scale confirmation of the reports using clinical data, (b) providing the community with size surrogate data for the head region which was not provided in the original reports, and additionally providing the measurements of patient ellipticity ratio for different body regions.

**Method:**

A total of 884 routine scans were included in our analysis including data from the head, thorax, abdomen, and pelvis for adults and pediatrics. We calculated the ellipticity ratio and all of the size surrogates presented in AAPM Reports 204/220. We correlated the purely geometric‐based metrics with the “gold standard” water‐equivalent diameter (D_W_).

**Results:**

Our results and AAPM Reports 204/220 agree within our data's 95% confidence intervals. Outliers to the AAPM reports’ methods were caused by excess gas in the GI tract, exceptionally low BMI, and cranial metaphyseal dysplasia. For the head, we show lower correlation (R^2^ = 0.812) between effective diameter and D_W_ relative to other body regions. The ellipticity ratio of the shoulder region was the highest at 2.28 ± 0.22 and the head the smallest at 0.85 ± 0.08. The abdomen pelvis, chest, thorax, and abdomen regions all had ellipticity values near 1.5.

**Conclusion:**

We confirmed AAPM reports 204/220 using clinical data and identified patient conditions causing discrepancies. We presented new size surrogate data for the head region and for the first time presented ellipticity data for all regions. Future automatic exposure control characterization should include ellipticity information.

## 1 INTRODUCTION

Dose from computed tomography (CT) has always been a general concern in the medical community.^1^, ^2^ This is primarily due to the growing number of CT examinations^3^ and the high dose from CT relative to other imaging modalities.^2^, ^4^ It is always a challenge for radiologists and medical physicists to establish adequate image quality with the lowest radiation exposure to the patient, in agreement with the ALARA (As Low As Reasonably Achievable) principle.^5^ Unfortunately, in CT, the current scanner output dose metrics, such as volume CT dose index (CTDI_vol_), do not reflect the dose the patient actually receives.^6^, ^7^, ^8^ The CTDI_vol_ only represents the system's radiation output for a very specific set of conditions in a cylindrical acrylic polymethyl methacrylate (PMMA) phantom with diameters of 16 or 32 cm in a contiguous axial or helical examination.^4^, ^7^, ^9^, ^10^, ^11^, ^12^ Ideally, a method would exist to normalize these dose values to make them reflect the dose a patient actually receives.

The American Association of Physicists in Medicine (AAPM) Report 204^12^ introduced the concept of a size‐specific dose estimate (SSDE). The SSDE is a patient size‐corrected estimate of patient dose which uses a surrogate for patient size to scale the scanner‐reported CTDI_vol_.^12^ Many previous studies have used and/or evaluated size surrogates to estimate patient size which include body weight, body mass index (BMI), age cross‐sectional diameter, effective diameter, and a combination of these parameters for individual dose adaptation for adults.^13^, ^14^, ^15^, ^16^, ^17^, ^18^, ^19^, ^20^, ^21^, ^22^, ^23^ AAPM Report 204 details the use of multiple size surrogates to normalize CTDI_vol_ values to SSDE including: anterior–posterior (AP) dimension, lateral (LAT) dimension, AP + LAT, circumference, and effective diameter $\sqrt{AP\times LAT}$. The methods of AAPM Report 204 have been evaluated for clinical adult and pediatric CT scans of the torso and truncated axial images.^8^, ^24^, ^25^, ^26^, ^27^ The size surrogates of AAPM Report 204, however, are based only on patient geometry and do not consider the different attenuation of various tissue types. For example, the lung was considered a caveat^28^ because of its much lower density compared to water or PMMA, therefore reducing the attenuation of the patient's chest significantly from the 32 cm reference CTDI_vol_ phantom.

This limitation was addressed in the AAPM Report 220^29^ in detail, and the sole use of water‐equivalent diameter (D_w_), which considers tissue attenuation in addition to patient geometric size, for calculations of SSDE is recommended. The use of D_W_ had previously been proposed before AAPM Report 220.^13^, ^18^, ^30^, ^31^ Wang et al.^30^ demonstrated that the use of D_W_ is more accurate in calculating SSDE in thoracic CT compared to the geometric size surrogates, but D_W_ and the geometric size surrogates both perform and correlate well for the abdomen and pelvis. AAPM Report 220 collected experimental data acquired using cylindrical phantoms and Monte Carlo simulations. The analysis assumed that the collection of a limited number of different size elliptical phantoms and the family of Monte Carlo phantoms used was intended to span what is seen clinically. Ikuta et al.^25^ evaluated D_E_ and D_W_ and found good correlation; however, their method differed from AAPM Report 220 where they used four slices separately corresponding to the lung apex, the superior aspect of the aortic arch, the carina, and immediately superior to the diaphragm without averaging for thorax and abdomen. However, the AAPM 204/220 Reports allow the use of the center of the scan range calling it a “shortcut” relative to averaging a size surrogate over the entire scan range. Leng et al.^32^ demonstrate that while D_W_ varies along the patient z‐axis, the D_W_ measured at the center of the scan is highly correlated with SSDE calculated using an average of patient size taken over the entire scan range. They compared their results to Cheng^26^ who showed similar results using D_E_ instead of D_W_. Noferini et al.^27^ performed an analysis similar to Leng et al.,^32^ albeit they used D_E_ instead of D_W_, by comparing the SSDE conversion factors derived using the center of a scan range and the maximum and minimum conversion factors within 20 cm of the center. Noferini et al. found SSDE differences within this range of 10% occurred in approximately 60% of abdomen scans and 80% of chest scans.^27^ While these studies address SSDE for particular body regions, there still needs to be a study that confirms the D_W_ as an estimate for D_E_ using methods described in the AAPM reports for a large set of clinical data in all body regions. The family of Monte Carlo phantoms will likely not capture all clinical variations observed in the clinic. Identifying clinically possible relevant patient's body habitus conditions that negatively impact the conclusions of AAPM Reports 204/220 is one motivation for the current work.

There is also a need to present head data in the form of AAPM Reports 204 and 220. Head scans are one of the most common CT examination types.^33^ The original AAPM 204/220 Reports did not consider the head in their analysis. AAPM Report 204 asserted that they only considered the abdominal region with their phantom models and AAPM Report 220 only considered the abdomen and thoracic regions. While the literature has few prior studies applying AAPM Report 204/220 like concepts to the head,^34^, ^35^ no study explicitly analyzes head data using the methods presented in both AAPM Reports 204 and 220. McMillan et al.^34^ compared D_E_ to D_W_ for head models using Monte Carlo simulations and they measured the CT axial slice just superior to the eyes; albeit, they do not present detailed geometric head data in the manner of AAPM Report 204 and the focus of their work was on organ dose estimates. Anam et al.^35^ only uses D_W_ and does not report data for the geometric size surrogates discussed in AAPM 204. The presentation of head geometric and D_W_ data facilitating an augmentation to the methods of AAPM 204/220 is another motivation for the current work.

**Figure 1:**
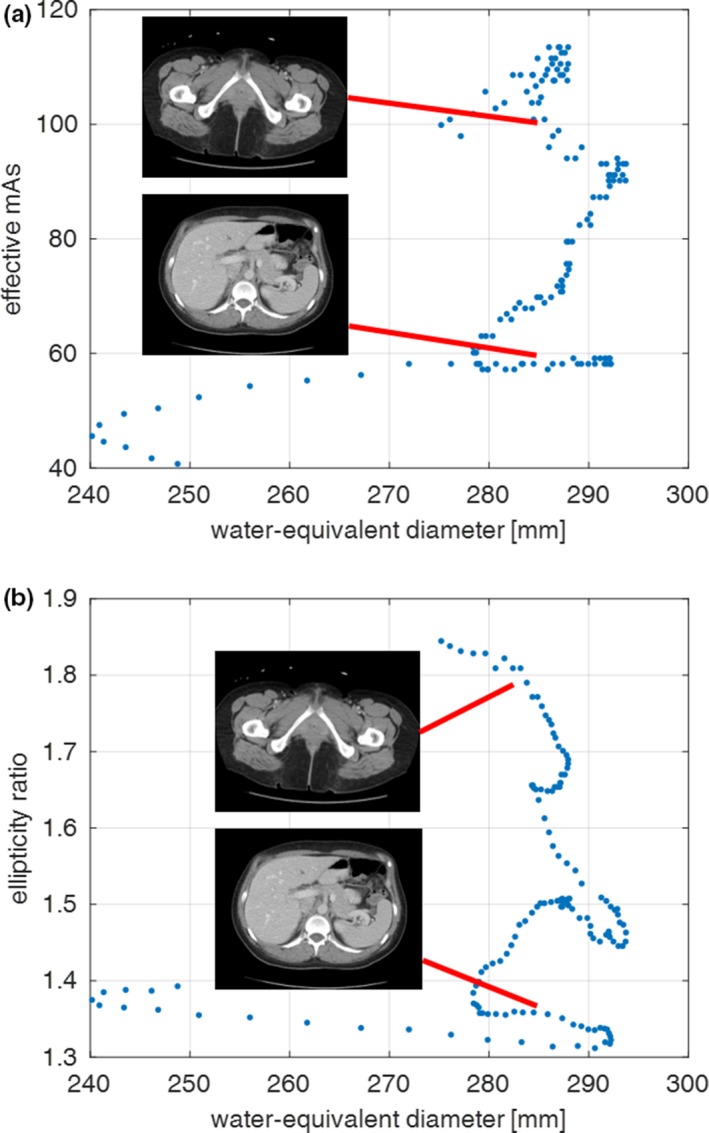
(a) Effective mAs as a function of water‐equivalent diameter (D_W_) along the z‐axis (cranial‐caudal), and (b) ellipticity ratio (LAT/AP) as a function of water‐equivalent diameter (D_W_) for the same patient as shown in a. For the same D_W_ of 283.6 mm, the mAs values are 105.7 and 58.2, and their ellipticity ratios are 1.79 and 1.36, respectively.

The methodologies of AAPM Reports 204/220 state that the center of the scan range can be used to calculate the geometric or D_W_ metric. Leng et al.^32^ demonstrate how this is an accurate way of estimating patient size relative to averaging over the entire patient's scan range for abdomen scans. The study of Leng et al. was needed since patients vary in size over the z‐axis dimension and automatic exposure control (AEC) systems vary scanner output over the z‐axis dimension; therefore, assuming that D_W_ at the center of the scan range models, the entire scan range was not well supported until Leng et al.'s work. Varying scanner output along the scan range is referred to as z‐axis dose modulation and it has been reported on extensively.^5^, ^36^, ^37^, ^38^, ^39^, ^40^ In addition to z‐axis modulation, some CT scanners can also vary the dose angularly about the patient.^6^, ^36^, ^37^, ^40^, ^41^, ^42^, ^43^, ^44^, ^45^, ^46^ Motivation for this comes from the patients’ cross sections not being perfectly circular. Details on how CT scanners modulate dose are not widely published; however, it is understood that the z‐axis modulation is related to the overall patient size (e.g., D_W_) and the angular modulation is related to the ratio of lateral to anterior–posterior thickness (LAT/AP). In other words, for a circular patient cross section, no angular dose modulation would be expected, while for highly elliptical cross sections, one would expect large angular dose modulation. Gies et al. showed that the angular dose modulation should be performed proportional to the square root of the patient's cross section in order to minimize image noise as a function of dose.^44^, ^45^ Giacomuzzi et al.^42^ demonstrated the clinical results from an angular dose modulation system, where CT localizer radiographs are acquired to estimate the patient thickness in the LAT and AP directions so that the tube current can be modulated sinusoidally around the patient. The study by Giacomuzzi et al.^42^ is the only study in the literature, to our knowledge, that reports the ratio of LAT to AP for patient scans, albeit for a relatively small number of patients. Recently, in our laboratory, we did not observe the expected exponential relationship between effective mAs ($\frac{mAs\;\times \;rotation\;time}{\mathrm{pitch}}$) and D_W_ when looking at effective mAs per image slice. This relation is expected when using an AEC system that is designed to keep image noise constant. We hypothesized that this was caused by the changes in the ratio of LAT/AP, also called ellipticity ratio (Szczykutowicz et al. “Not all water equivalent diameters yield the same dose: The influence of patient ellipticity on AEC algorithms in CT” presented at the 2016 annual meeting of the Radiological Society of North America SSE21‐05). Giacomuzzi et al.^42^ explicitly demonstrate in Fig. 3 of their paper that ellipticity ratio influences the dose reduction amount when angular dose modulation is used.^42^ Figures 1(a) and 1(b) demonstrate that when two patients’ image slices had the same D_W_ of ~283.6 mm, their measured effective mAs values were 105.7 and 58.2. These image slices corresponded to large differences in ellipticity value 1.79 and 1.36, respectively. The data shown in Fig. 1 were collected using the scan parameters listed in Table 1 for the routine adult abdomen pelvis dataset which used angular dose modulation.

**Table 1:** Experimental data collection of human patients of routine adult abdomen and pelvis, adult chest, adult head, and pediatric abdomen pelvis cases (the pediatric data included five different protocols hence the range in NI, pitch, and slice thickness). ^†^Denotes datasets that are derived from the adult chest dataset scan range. The Noise Index (NI) refers to a vendor‐specific automatic exposure control setting. Other vendor‐specific reconstruction options were set as follows: “PLUS” mode, recon kernel of “STANDARD” for the body and “SOFT” for the head, and an ASiR level of 40%

Data set	kV	Noise index	Pitch	Slice thickness (mm)	Interval (mm)	Scan range
Adult abdomen pelvis	120	15.5	0.516	5	3	Above dome of liver to pubic symphysis
Adult chest	120	21.0	0.516	5	3	Above lung apices to below lung bases
Adult shoulder^†^	120	21.0	0.516	5	3	Superior 3 cm of chest scan range
Adult thorax^†^	120	21.0	0.516	5	3	Central 40% of scan range of chest data
Adult abdomen only^†^	120	21.0	0.516	5	3	Inferior 3 cm of chest scan range
Pediatric abdomen pelvis	80	12.0–17.0	1.375–0.516	3.75–2.25	2.25	Above dome of liver to pubic synthesis
Adult head	120	3.4	0.531	5	3	From maxilla region to over the top of skull

The ellipticity ratio is involved in setting the angular dose modulation value; however, there is only one paper reporting ellipticity values to our knowledge in the literature.^42^ Therefore, in this paper, we report the ratio of LAT to AP for multiple body regions, including the head for hundreds of patients. We do not report on how this value influences a CT scanners’ dose modulation since that is highly vendor dependent and “black box” in nature. However, there are several papers in our field that are actively “reverse engineering” vendors AEC algorithms for research and clinical purposes.^47^, ^48^, ^49^ The ellipticity data we report here can be included in such efforts.

As motivated in the previous paragraphs, the purpose of this paper is to confirm AAPM reports 204/220 and provide data for the future expansion of these reports by: (a) presenting the first large‐scale confirmation of the reports using clinical data, (b) providing the community with size surrogate data for the head region which was not provided in the original reports and additionally provide the measurements of patient ellipticity ratio for different body regions.

## 2 METHODS

### 2.A Experimental data collection

**Figure 2:**
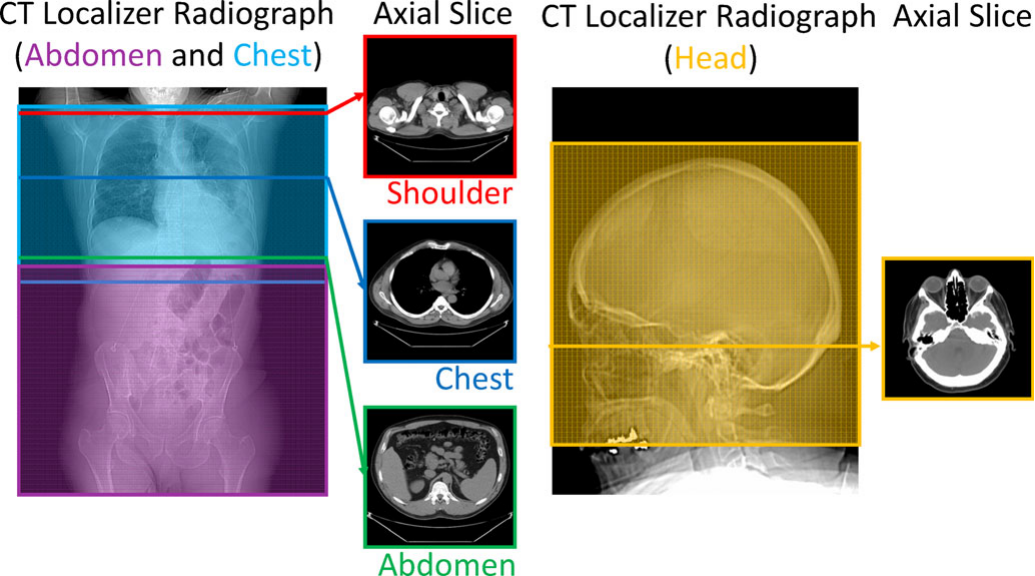
Scan range of adult and pediatric abdomen pelvis (purple), adult chest (cyan), and adult head (orange) scans shown on a CT localizer radiograph. The adult chest scan is broken up into three subsets to produce a total of six datasets. Examples of single axial CT slices of these subsets for shoulders (red), thorax (blue), and abdomen only (green) are shown as well as an example head axial slice (orange).

A total of 884 patients were included in our analysis. The patients’ data were collected from three different examination types and binned into six different sets. Table 1 shows the four main CT datasets: (a) routine adult abdomen and pelvis scans (297 patients total), (b) chest scans (300 patients total) with chest data separated into shoulder, thorax, and abdomen, (c) pediatric abdomen pelvis scans (87 patients total), and (d) adult head scans (200 patients total). Figure 2 shows an example of a CT localizer radiograph for one patient and it shows the scan range for adult abdomen and adult chest with the examples of single CT axial slices from subset scan regions of the shoulder, thorax, and adult abdomen only.

Table 1 provides information about the data collected. All data were acquired from 64 slice CT scanners from the same manufacturer (Optima CT660 and Discovery HD750 CT scanner models from GE HealthCare, Chicago, IL, USA). All data were collected under an IRB‐approved protocol in a retrospective manner in which the patient consent was waived. For all protocols, automatic exposure control (AEC) was turned on. For each patient, the beginning and end of each of the scan ranges were determined on the basis of anatomic markers: chest scan ranges were from just above the lung apices to just below the lung bases and abdominal pelvis scan ranges were from just above the top of the liver to the pubic symphysis. Therefore, the adult chest data contain images of the shoulder region and the abdomen region, and the adult abdomen pelvis data contain images of the thorax region. This slight overlap in scan regions is needed in the clinic to ensure adequate coverage of the desired body region. The overlap and scan ranges for abdomen, chest (with examples of single axial CT slices of shoulders, thorax, and abdomen only), and head (with single axial CT slice) are illustrated in Fig. 2. For this study, we look at all datasets in their entirety, using all axial images slices from the entire scan range, and we analyze the subsets of the scan ranges. The motivation for this is due to chest scans containing some of the shoulders at the superior end of the scan range and part of the abdomen at the inferior end of the scan range. One would expect different relationships to exist between scanner output and size surrogates depending on the body region, which is why we analyzed specific regions. The pediatric abdomen pelvis, adult head, and adult abdomen pelvis datasets were not broken into subsets for analysis.

### 2.B Effective Diameter, LAT, AP, and ellipticity calculation

AAPM Report 204 describes that the lateral (LAT) and anterior–posterior (AP) dimensions can be determined from the CT image.^12^ We performed two image processing steps to the axial images before measuring the AP and LAT dimensions. First, we applied a threshold to the image set at −150 HU which set all pixels with values greater than this threshold to 1 and less than or equal to the threshold to 0. Second, we used connected component analysis to remove any objects outside of the contours of the patient (e.g., the couch, EKG leads, tubing, and cloths/blankets). The component analysis (bwareaopen function from MATLAB, the Mathworks INC, Natick, MA, USA) kept all structures with more than 10 #bib000 connections to create the binary image. We then extracted the maximum width and height of the binarized patient cross section to obtain the LAT and AP patient dimensions respectively. The effective diameter, denoted here as D_E_, is the diameter of a circle containing the same cross‐sectional area as that of an ellipse with axes defined by the AP and LAT dimensions of the patient. AAPM Report 204 provides a derivation of D_E_ and we provide the final solution

(1) $$D_{E}=\sqrt{\mathrm{AP}\times \mathrm{LAT}}$$

For the purpose of analysis, we calculate the AP, LAT, and D_E_ for each slice in each dataset and then report the average for all slices for each patient or each subset of patient data as defined in Table 1.

We define the ellipticity ratio as r = LAT/AP. The variable r is calculated for every slice and then averaged overall slices in a given dataset for each patient as described in Table 1. We also report the standard deviation in r and the minimum and maximum r values observed for each dataset shown in Table 1. AAPM 204 uses a second‐order fit to relate D_E_ to AP or LAT. The authors of AAPM 204 use a first‐order fit to relate D_E_ to AP + LAT. We believe the reason that a second‐order fit gave a better result for AP or LAT was due to the phantoms used in the AAPM study. For a fixed ellipticity ratio, D_E_ should be proportional to AP or LAT. The relationship between D_E_ and LAT (or AP with a simple substitution using r = LAT/AP) is

(2) $$D_{E}=\sqrt{k}\times \text{LAT}$$

where k = 1/r. In other words, for a fixed ellipticity ratio, a first‐order fit should be adequate to relate D_E_ to LAT or AP. The AAPM 204 report, however, includes cylindrical phantoms (r = 1) and some elliptical phantoms of a fixed r but varying size. This is why we believe the authors used a second‐order fit between D_E_ and AP or LAT. Not because the underlying relationship between D_E_ and AP or LAT warranted this, but because the combination of varying r values made their data nonlinear. Therefore, we chose to use a first‐order fit of our clinical data since it includes hundreds of patients with varying r values. We assumed a given body region in a human would have a distribution of r values with a mean that would be characteristic of that body region. Furthermore, as seen in our results, the second‐order fits of AAPM 204 phantom data fall within our confidence intervals.

### 2.C Water‐equivalent diameter

Previous studies show the x‐ray attenuation of a patient in terms of a water cylinder with a water‐equivalent diameter (D_W_).^12^, ^13^, ^30^, ^31^, ^32^, ^35^, ^36^, ^50^ In other words, the D_W_ represents the diameter of a cylinder of water that contains the same total x‐ray attenuation as that contained within the patient's axial cross section and depends on both the cross‐sectional area of the patient and the attenuation of the contained tissues. This method of calculating D_W_ was described in AAPM Report 220 and implemented it here with equation

(3) $$D_{W}=2\sqrt{\left[\frac{1}{1000}\bar{\mathrm{ROI}}+1\right]\frac{A_{\mathrm{ROI}}}{\pi }}$$

The ROI represents the mean CT number within the reconstructed field of view (FOV), and A_ROI_ is the product of the number of pixels in the ROI and the pixel area. Our ROI was inscribed inside the reconstructed DICOM images for each patient. Since the DICOM images are square matrices, we inscribed a circle inside each DICOM image with a diameter equal to the entire width of the image. In some cases, when the reconstructed image center was not at isocenter, this ROI could contain “padding” values of −3024 HU. Therefore, we applied a remapping of all of the values inside the circle used to calculate the mean CT number which mapped all signals equal to −3024 to −1000 HU to simulate air. The use of “padding” values is common to most CT vendors, but the “padding” value may differ. Failure to correct for this would decrease the D_W_ values. We did not perform any thresholding or connected component analysis of the axial image data prior to calculating D_W_. For analysis, we calculate D_W_ for each slice in each dataset and then report the average for all slices for each patient or each subset of patient data as defined in Table 1.

### 2.D Data analysis

AAPM Report 204 reported D_E_ as function of AP + LAT, LAT, and AP to see if all three could be used to estimate the patient dose using a Monte Carlo (MC) or MC‐derived patient dose calculation and using images of scanned cylindrical phantoms. Here, we used the same approach, but with clinical data, and we included the AAPM Report 220 patient surrogate D_W_. For all datasets, we plotted D_W_ versus D_E_. For just the head dataset, we plotted D_W_ versus DE, AP, LAT, (AP+LAT)/2. We separated the adult chest into three regions corresponding to the shoulders, thorax, and abdomen only as shown in Table 1 and plotted D_W_ as a function of D_E_ for each subset. All plots were fitted using a linear fitting routine (*polyfit* function from MATLAB, the Mathworks INC, Natick, MA, USA). We applied a first‐order linear fit and linear regression (R^2^) to all data points combined and 95% confidence intervals for all data points. A 95% confidence interval indicates that a 0.95 probability of data points contain the true population mean. We report the confidence interval in millimeters and this number is the distance from the trend line to the confidence interval, so the range between confidence intervals is double the reported confidence interval in millimeters. We considered points outside this confidence interval to be outliers and we analyzed each of them to characterize deviations from the correlation shown in the AAPM reports that may be present in the clinic.

We plot the lines of best fit reported in AAPM Report 204 and, separately, plot the data obtained in 220 to see if their phantom and simulated predictions match our clinical data. AAPM Report 204 provided fitting details between D_E_ and the other geometric size surrogates AP, LAT, and AP + LAT, and we use this to directly compare the lines of best fit with 95% confidence we achieve with our clinical data. The AAPM Report 220 provides tables (Tables 1 and 2 of the AAPM Report 220) of D_E_ to D_W_ for a range of phantom sizes. The AAPM Report 220 lists results for abdomen (AAPM Report 220 Table 1, titled: Effective Diameter: $\sqrt{LAT\times AP}$) and thorax (AAPM Report 220 Table 2, titled: Water Equivalent Diameter: From CT Image). To compare to the AAPM abdomen data, we used the fit trend lines from our adult abdomen pelvis and pediatric abdomen pelvis data, and to compare the AAPM thorax data, we used the fit trend lines from our adult thorax dataset.

**Table 2:** Elliptical ratio (LAT/AP) calculation for patients of routine adult (abdomen and pelvis), adult chest (^†^subset of adult chest), and pediatric cases

Data set	Mean ellipticity ratio (LAT/AP)	Std. Dev.	Min–Max
Adult abdomen pelvis	1.48	0.22	1.20–1.94
Adult chest	1.60	0.23	1.21–2.07
Adult shoulder^†^	2.28	0.22	1.08–3.34
Adult thorax^†^	1.51	0.21	1.09–1.98
Adult abdomen only^†^	1.38	0.20	1.16–1.73
Pediatric abdomen pelvis	1.53	0.30	1.07–1.75
Adult head	0.85	0.08	0.83–0.87

## 3 RESULTS

### 3.A D_E_ vs D_W_ for clinical data

**Figure 3:**
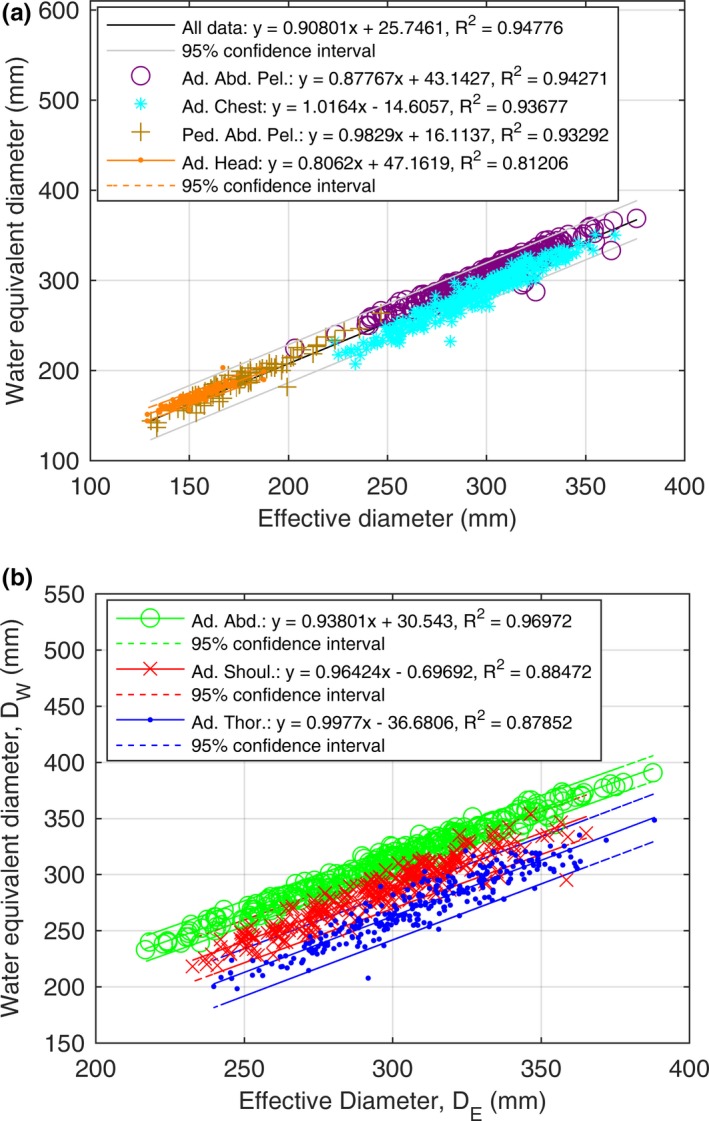
Correlation of D_W_ and D_E_. (a) The all data trend line includes the adult abdomen pelvis, the pediatric abdomen pelvis, and the adult chest and excludes the head (plotted separately). (b) From the adult chest dataset, the adult thorax, adult shoulder, and adult abdomen only data subsets are also shown.

Figure 3(a) shows the correlation of D_W_ as a function of D_E_ (R^2^ = 0.94776, 95% confidence interval range of ~23 mm) for all data excluding the head making D_E_ a reliable estimate of D_W_ for a large majority of cases, as expected. Separately, the adult abdomen pelvis (R^2^ = 0.94271), the adult chest (R^2^ = 0.93677), and the pediatric abdomen pelvis (R^2^ = 0.93292), and adult head data (R^2^ = 0.81206, 95% confidence interval range of ~8 mm) all show good correlation with the exception of the head data that have an R^2^ = 0.81206. Figure 3(b) shows the shoulder, thorax, and abdomen of the chest scan separately and each maintain good correlation between D_W_ and D_E_, abdomen (R^2^ = 0.93972) and shoulders (R^2^ = 0.88472) and thorax (R^2^ = 0.87873).

### 3.B AAPM 204/220 comparison

**Figure 4:**
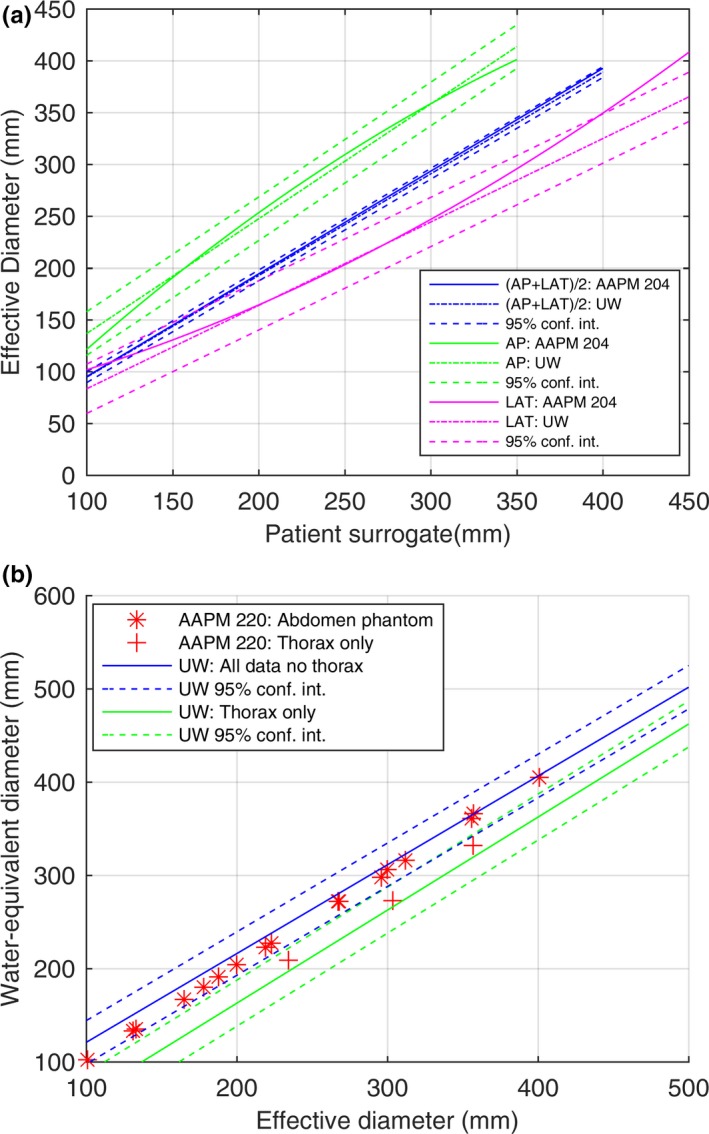
(a) Comparison of AAPM Report 204 for D_E_ as a function of patient size surrogate: (AP + LAT)/2 (blue lines), AP (green lines), and LAT (magenta lines) for AAPM Report 204 fits (solid lines), UW first‐order fit (dotted‐dashed lines) and 95% confidence interval (colored dashed lines). (b) Comparison of AAPM Report 220 D_W_ as a function of D_E_ calculated our fit for pediatric and adult abdomen pelvis data (blue) with 95% confidence intervals (blue dashed line) and our fit for adult thorax only (green) with 95% confidence intervals (green dashed line). In (b), AAPM Report 220 points for abdomen (red asterix) and thorax (red plus sign) are plotted over our fits.

Figure 4(a) shows both our work at the University of Wisconsin–Madison (UW) and AAPM Report 204 fits of D_E_ as a function of patient size surrogates, AP, LAT, and (AP + LAT)/2, for all data excluding the head. The AAPM fit falls well within our 95% confidence interval despite the AAPM use of a second‐order polynomial fit. Figure 4(b) shows both the UW fit of D_W_ as a function of D_E_ with data points taken from AAPM Report 220 Table 1 for abdomen and Table 2 for thorax, and shows that these points fall within our 95% confidence interval.

### 3.C D_W_ vs AP, LAT, (AP+LAT)/2 for adult head

**Figure 5:**
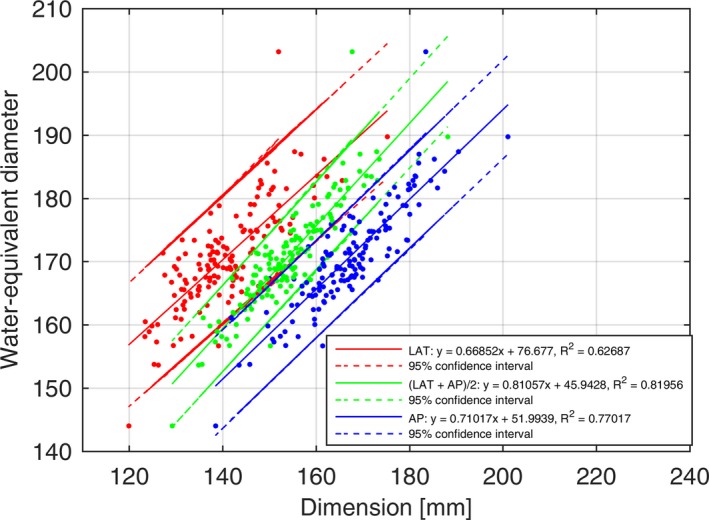
Correlation of LAT, AP, and (LAT + AP)/2 with D_W_ for adult head.

Figure 5 shows that D_W_ has relatively poor correlation as a function of AP (R^2^ = 0.77017, 95% confidence interval range of ~10.5 mm), LAT (R^2^ = 0.62687, 95% confidence interval range of ~15.1 mm), and (AP + LAT)/2 (R^2^ = 0.81956, 95% confidence interval range of ~8.1 mm).

### 3.D Ellipticity values

Table 2 reports the mean, standard deviation, and the minimum and maximum ellipticity values for all six datasets analyzed in our study. As one would expect, the ellipticity value is highest for the shoulder region (r = 2.28 ± 0.22). The head is the only body region we analyzed with an ellipticity value under unity (r = 0.85 ± 0.08) and also had the smallest standard deviation and minimum to maximum range. The average for all body regions minus the shoulders and head was approximately 1.5 for our patient population.

## 4 DISCUSSION

For all data excluding the head, we show in Fig. 4(a), that our linear fits of D_E_ as a function of (AP + LAT)/2, LAT, and AP compare well to the results of AAPM Report 204. For D_E_ as a function of AP or LAT as shown in Fig. 4(a), we did not observe the same curvature as AAPM Report 204; however, the spread in our data, shown by the 95% confidence interval, could have been hiding such behavior. As reported in AAPM Report 204, the physical phantoms used were from Boone et al. and Strauss et al., and both had circular cross sections (no elliptical shape, r = 1.0) whereas the Monte Carlo Voxelized Phantoms used by ICRU92 were elliptical. As discussed in our methods section, D_E_ is inversely proportional to the square root of the ellipticity factor when plotted against LAT. We used patient scans to do our analysis, which had a range of ellipticity values as shown in Table 2. Only the LAT comparison from the AAPM Report 204 data is outside our 95% confidence interval for patient LAT dimensions over 400 mm. In Fig 4(b), all AAPM Report 220 data points lie within our 95% confidence intervals for both the abdomen and thorax AAPM data. We obtained results agreeing with the phantom‐based results of AAPM Reports 204 and 220 using a large set of patient data. Our dataset is the largest clinical dataset used for this purpose to date and has allowed us to identify a number of outlier cases not previously reported on in the literature.

**Figure 6:**
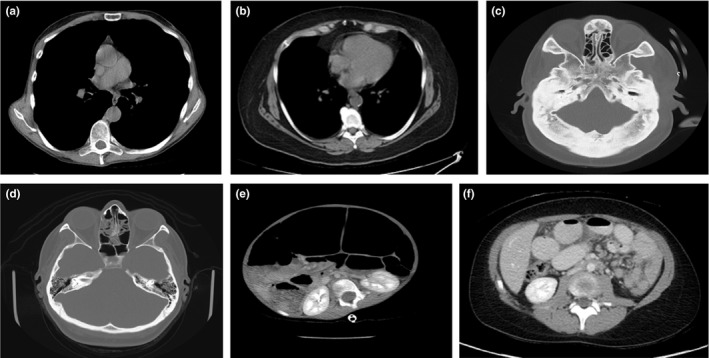
Examples of normal and outlier cases. The chest outlier (a) has a higher ratio of lung tissue to soft tissue and less subcutaneous fat relative to a typical chest scan (b). The head outlier (c) patient suffers from metaphyseal dysplasia (excess bone in the head) which increased the D_W_ relative to a typical head scan (d). The pediatric outlier (e) has excess gas in their GI tract causing their D_W_ to decrease relative to a typical pediatric patient (f).

There were a few outlier cases that deviated from our fits and the correlation shown in the AAPM task group reports. Figure 6 displays the outliers seen in Fig. 3(a) at the following locations: pediatric outlier at D_W_ = 181 mm and D_E_ = 200 mm, head outlier at D_W_ = 203 mm and D_E_ = 168 mm, and an adult chest outlier at D_W_ = 232 mm and D_E_ = 275 mm. Figure 6 shows example outlier and normal cases. Figure 6(e) shows the pediatric abdomen outlier where the D_W_ value is shown to be 20 mm below the fit line in Fig. 3(a) and this is due to the excessive amount of air gas in the gastrointestinal tract and the relatively lower amount of subcutaneous fat relative to other pediatric abdomen patients (e.g., compare to Fig. 6(f)) of the same geometric size. The adult chest outlier shown in Fig. 6(a) corresponds to the adult chest outlier in Fig. 3(a) which was also 20 mm below the fit line. This is due to the relatively higher ratio of lung space to soft tissue in the thorax to other adult chest scans (e.g., compare to Fig. 6(b)) and relatively lower amount of subcutaneous fat relative to other adult chest patients of the same geometric size. The head outlier case shown in Fig. 6(c) was 23 mm above the fit line for all head scans. The head outlier case presents with cranial metaphyseal dysplasia (excess bone in the head), which when compared to a “normal” adult head (i.e., compare to Fig. 6(d)), it is obvious that the excess bone is the reason for the higher D_W_ relative to other heads of the same geometric size. We do not show the adult abdomen pelvis outliers that can be seen in Fig. 3(a). We analyzed these cases and noted that these cases were always below the fit line, corresponded to cases that included more of the thorax region than was typical for a routine abdomen pelvis scan. Clinically, this is warranted in some cases when: (a) a radiologist requests coverage into the thorax or (b) for patients with lung bases that extend deep within the abdomen or conversely a diaphragm/liver dome that extends deep within the thorax. Therefore, when one scans an abdomen pelvis and includes more of the lungs than is typical for such a scan, D_W_ will decrease.

Ikuta et al. compare D_W_ to D_E_ for the thorax and abdomen and report poor (R^2^ = 0.51) and good (R^2^ = 0.90) correlation in those regions, respectively. 25 Our correlation coefficients are much higher than the Ikuta result. We believe that the source of this difference is sample size. We analyzed on average 110 image slices for each of our chest datasets whereas Ikuta looked at 50 patients and measured four slices per patient. The four slices corresponded to the lung apex, the superior aspect of the aortic arch, the carina, and immediately superior to the diaphragm. Ikuta et al. reported fitting statistics not on the average of their four measurements per scan, but for each measurement point individually. If we compare D_W_ and D_E_ for each point in our chest dataset individually (not plotted in this paper) and perform no examination averaging, our correlation coefficient drops from 0.937 to 0.589 for the adult chest data. This can be understood by looking at Fig. 3(b), the four measurement points taken by Ikuta et al. span the three different anatomical regions within a routine chest scan, the shoulders, thorax, and abdomen. These regions, for the same geometric size, do exhibit relatively large differences in D_W_.

For the chest relative to the abdomen, we expected the D_W_ to be much lower because of the thorax (air‐filled regions of the lung).^28^ We examined a few adult chest patients’ scans and noticed that the shoulders and abdomen were included and it is necessary to include them in a routine adult chest procedure in order to ensure the lung apices and bases are covered. We separated the chest region into subset regions of adult shoulders, adult thorax, and adult abdomen only, shown in Fig. 3(b) and we observed a clear distinction of D_W_ for all subsets. Both adult abdomen only and adult shoulder have a higher D_W_ than the thorax region where the abdomen and shoulders, on average, are approximately 55 and 27 mm higher than the thorax, respectively. The combination of higher D_W_ from shoulders and adult abdomen only increases the adult thorax D_W_ by approximately 20 mm. Combining this observation with the comments about some of the abdomen pelvis outliers being due to including more of the thorax motivates a general takeaway from our results. This takeaway is that the contributions from all body regions included within an examination must be considered when discussing patient size surrogates. This is especially true since x‐ray attenuation will change drastically as one moves from the abdomen to the thorax and from the mid‐thorax up into the lung apices (e.g., and moves into the shoulders).^32^ At such boundaries between patient body regions, vendors’ AEC algorithms are likely to greatly change the tube output.

We were also surprised to notice that the adult shoulder data appeared to have a much lower D_W_ than the abdomen for the same D_E_ as shown in Fig. 3(b). Looking at the adult shoulder data is clinically relevant as this body region corresponds to cervical spine imaging, neck CTA imaging, and shoulder imaging. The shoulders are also included in the scanning of other body regions like the chest as shown in the present analysis. One would expect the shoulders to have a higher D_W_ relative to the abdomen for the same D_E_ because of bony anatomy of the shoulders and arms. Albeit, some air‐filled regions could also present due to the lung apices. However, we found that the D_W_ for the shoulders is shifted to the right (e.g., decreased D_W_ value) because the adult shoulders’ LAT dimension is relatively larger compared to the adult thorax and adult abdomen only, and Table 2 shows that the ellipticity for the adult shoulders to be quite high (r = 2.28 ± 0.22). The high ellipticity of the shoulders causes the D_W_ for the adult shoulders to appear lower than the abdomen for the same geometric size surrogate, D_E_. In other words, for the same LAT measurement on an abdomen pelvis and shoulder scan, the AP would be much larger for the abdomen pelvis relative to the shoulders.

For the head, we related D_W_ to LAT, AP, and (AP + LAT)/2 in Fig. 5 and D_W_ to D_E_ in Fig 3(a). We found poor correlation between the size surrogates for the head overall. We noted that our image processing steps for obtaining the geometric size‐based metrics AP and LAT (from which D_E_ is derived) included the ears and nose. Therefore, for patients with their ears protruding far from their head, the LAT measurements would increase, predicting the patient was more attenuating than we would desire for the purposes of SSDE calculations. We noticed the same behavior for the nose and the AP length calculation. We also noted that the angle of the head (defined by a line connecting the orbits and ear cannel, e.g., the orbital‐meatal line) varied patient to patient and effected AP and LAT measurements. We confirmed that we were able to remove the head holder and couch from the geometric size measurements of AP and LAT, so size contributions from these non‐patient objects were not present in our data. We confirmed the head holder and/or couch was not present in AP and LAT length calculations by manually reviewing the thresholded and segmented axial images described in Section 2.B. The relatively poor correlation (R^2^ = 0.81206) for D_W_ vs D_E_ for the adult head scan in Fig 3(a) was not surprising considering the correlation was similar to the one in the work by McMillan et al. (R^2^ = 0.87). In their work, they used the slice above the eyes (e.g., a single slice) differing from our use of the entire head scan range which could explain their slightly better correlation coefficient. Another external comparison of our data can be made to that of Aman et al.^35^ Anam et al. show D_W_ values for 17 head patients. Anam et al. obtained D_W_ at the level of the orbits and found an average of 181 ± 6.6 mm. The average of our head data provided a D_W_ of 171 ± 7.9 mm. We looked into why our measurement was reporting lower values relative to the Anam study. One would expect the Anam result to have lower D_W_ than our results since the Anam study removed the CT couch from the measurement while we did not. Anam et al. found that by removing the couch, for head scans, the D_W_ decreased on average by 1.54%. We believe that the source of the difference in our values being smaller than Anam was because we averaged the D_W_ over the entire head scan range. Anam took their measurements at the widest point of the head, the orbits. Our D_W_ measurements were taken over the entire head which would decrease our D_W_ value.

As stated in the introduction, patient ellipticity is incorporated into CT vendor's angular dose modulation systems. Giacomuzzi et al.^42^ showed that as the ellipticity ratio changed from 2.7 to 1.6, the dose reduction amount with angular dose modulation decreased from approximately 18% to 11%. To our knowledge, this is the only work detailing such AEC behavior as a function of patient ellipticity. To understand how this will influence a given CT scanner's performance, detailed vendor‐and scanner‐specific modeling is required. McMillan et al. recently performed such a characterization which modeled the angular dose delivery of a CT scanner.^49^ To augment McMillan's work, and future works like it, our results provide the community with the first set of ellipticity data reported for multiple body regions including adults and pediatrics. Our results demonstrate that the ellipticity ratio changes for different body regions as expected as shown in Table 2. Our results also demonstrate that within routine scan ranges like CT scanning of the chest, the ellipticity ratio will vary as evident by comparing the ellipticity ratio of the shoulders, thorax, abdomen only, and chest in Table 2. Leng et al.^44^ show that since automatic exposure control (AEC) is widely used in most torso and head CT scans, tube current and consequently CTDI_vol_ also change with patient size. Therefore, both components of SSDE, the conversion factor and CTDI_vol_, change with varying size and attenuation along the z‐axis within any given patient. While Leng et al. show that this variation in size adjustment factor for SSDE calculations can accurately be measured by just using the central slice of a scan range, accurate knowledge of scanner mA would require ellipticity ratio information over the entire scan range. This observation is supported by Table 2 and noting that for the chest scan region, for example, the ellipticity ratio goes from 2.28 over the shoulders to a low of 1.38 over the abdomen. Knowledge of body region–specific angular tube current modulation due to ellipticity ratio would be needed for organ‐specific dose calculations, of which SSDE is not.

We recommend that the user individually measures LAT and AP dimensions and does not use the ellipticity values reported in Table 2 to determine the LAT or AP dimension given an AP or LAT measurement, respectively.

One limitation of our study is that we did not relate our patient size surrogates directly to dose as other studies have done, and although this was not the purpose of this study, it is important to note. Such a comparison will be highly vendor dependent, as each vendor's AEC implementation will respond differently to the size surrogates presented in this paper and additionally to other influences like patient ellipticity and geometric magnification. We did not remove the couch or head holder when calculating Dw. We think that this is fine because Anam et al.^35^ show that the effect of the table (e.g., couch) is on the order of 1.5%–6.2% (smallest for head scans and largest for chest scans). We chose not to complicate our calculations of D_W_ with more image processing steps for this reason. We also did not investigate further methods for removing the influence of the ears and/or nose on head AP and LAT calculations. No other works in the literature have considered removing these features either.

Another limitation of our work is the difference in methodology for calculating D_W_ relative to AAPM Report 220. AAPM Report 220 explicitly states that the couch should be removed prior to calculating D_W_. We chose not to remove the couch. Removing the couch requires image processing. Such processing requires: (a) calculation time, which could be a factor if calculated on every image of every CT examination at an institution, (b) a model for the CT number, size, and relative position of the couch to the patient allowing for its segmentation, and (c) flexibility to handle different couches (bariatric couches, “regular diagnostic CT couches”, radiotherapy flat table tops, etc.). We implemented a couch removal algorithm for the geometric size surrogates. However, AAPM Report 220 states that D_W_ is the preferred method for SSDE calculation. We felt that differences in couch removal strategies could unnecessarily complicate SSDE calculation and/or bias their results if their performance differed. We feel confident that couch removal is actually not needed. Our results as shown in Fig. 4(b) agree with the AAPM Report 220 results. This agreement provides us confidence that a couch removal strategy is not required. Furthermore, Anam et al.^35^ demonstrated that the couch has minimal impact on D_W_.

## 5 CONCLUSION

Following AAPM Reports 204/220 using a clinical dataset containing 884 patients we made the following specific conclusions:

1. We identified sources of outliers in our data that deviate from the trend lines shown in AAPM Reports 204/220 including: medical conditions causing excess bone formation inside the skull (cranial metaphyseal dysplasia), lack of subcutaneous fat relative to others in the patient population (low BMI), and deviations from typical scan ranges for a particular examination type (e.g., including parts of the thorax in an abdominal pelvis scan).
2. We applied the methodologies of the size surrogates of AAPM Report 204 and AAPM Report 220 to different body regions and age groups including the head. The head has not previously been reported on using the framework of the AAPM Reports 204/220. Our fit lines for D_E_ and D_W_ for the abdomen and chest agreed with the AAPM 204 and 220 within our 95% confidence intervals.
3. For the first time to our knowledge, we report patient ellipticity values derived from clinical scans. We report values for adult chest, adult abdomen pelvis, adult head, pediatric abdomen pelvis, adult shoulder, adult thorax, and adult abdomen body regions. Such a description of patient form/shape will be needed to understand and reverse engineer some CT vendors “black box” AEC algorithms.

## IRB STATEMENT

All data were collected under an IRB‐approved protocol in a retrospective manner in which the patient consent was waived.

## CONFLICT OF INTEREST

TPS receives research support, is a consultant, and supplied CT protocols under a licensing agreement to GE Healthcare. TPS is the founder of protocolshare.org. CSB receives research support from GE Healthcare. For CSB there are no disclosures. TPS is on the MAB of iMALOGIX LLC.
